# A four-step protocol to overcome loops/tortuosity during transradial coronary interventions: introducing the ‘’Serpentine’’ technique

**DOI:** 10.1007/s12928-026-01251-9

**Published:** 2026-03-21

**Authors:** Grigorios Tsigkas, Nikoleta Kalovrenti, Spyridon Graidis, Michail Papafaklis, Dimitrios Chlorogiannis, Athanasios Moulias, Georgios Vasilagkos, Eleni-Evangelia Koufou, Panagiota Spyropoulou, Nikolaos Vythoulkas-Biotis, Nikolaos Kartas, Periklis Davlouros

**Affiliations:** 1 University of Patras, Cardiology Department, Rion, Patras, 26504 Greece; 2 University Hospital of Patras, Cardiology Department, Rion, Patras, 26504, Greece

**Keywords:** Loop, Tortuosity, Radial artery, ‘’Serpentine’’ technique

## Abstract

The radial artery access is frequently used in coronary interventions. However, anatomical variations, such as loops, can pose significant challenges, leading to an increased number of access site crossovers. This study presents a four-step protocol introducing the “Serpentine” technique, a novel approach based on specific maneuvers using the Tiger-II catheter to navigate radial artery loops. This was based on a prospective registry of patients who underwent transradial coronary angiography at our tertiary hospital between 2020 and 2022 and our predefined four-step protocol was applied. Procedural success was defined as the completion of coronary angiography without the need for access site crossover. The incidence of access site crossover was evaluated. A total of 2,389 patients underwent coronary angiography at our center during the study period, and 130 patients (5.44%) were found to have radial artery extreme tortuosity/loop, with a mean age of 74 years. Of these, 75 patients (58%) were men. Using our protocol, radial artery access was successfully achieved in 124 patients (95.4%) with a median total procedure time to successful initiation of coronary angiography of 60 s (IQR 30–120). Only 6 patients (4.6%) required crossover to an alternative access site, and no severe anatomical complications were observed. Success by protocol step was as follows: Step 1 in 48 (37%), Step 2 in 61 (47%), Step 3 in 10 (7.7%), and Step 4 in 5 cases (3.8%). The proposed four-step strategy represents a promising, safe, and effective algorithm for overcoming loops during transradial coronary angiography.

**Graphical abstract:**

Created in BioRender. Graidis, S. (2025) https://BioRender.com/z57kwul.

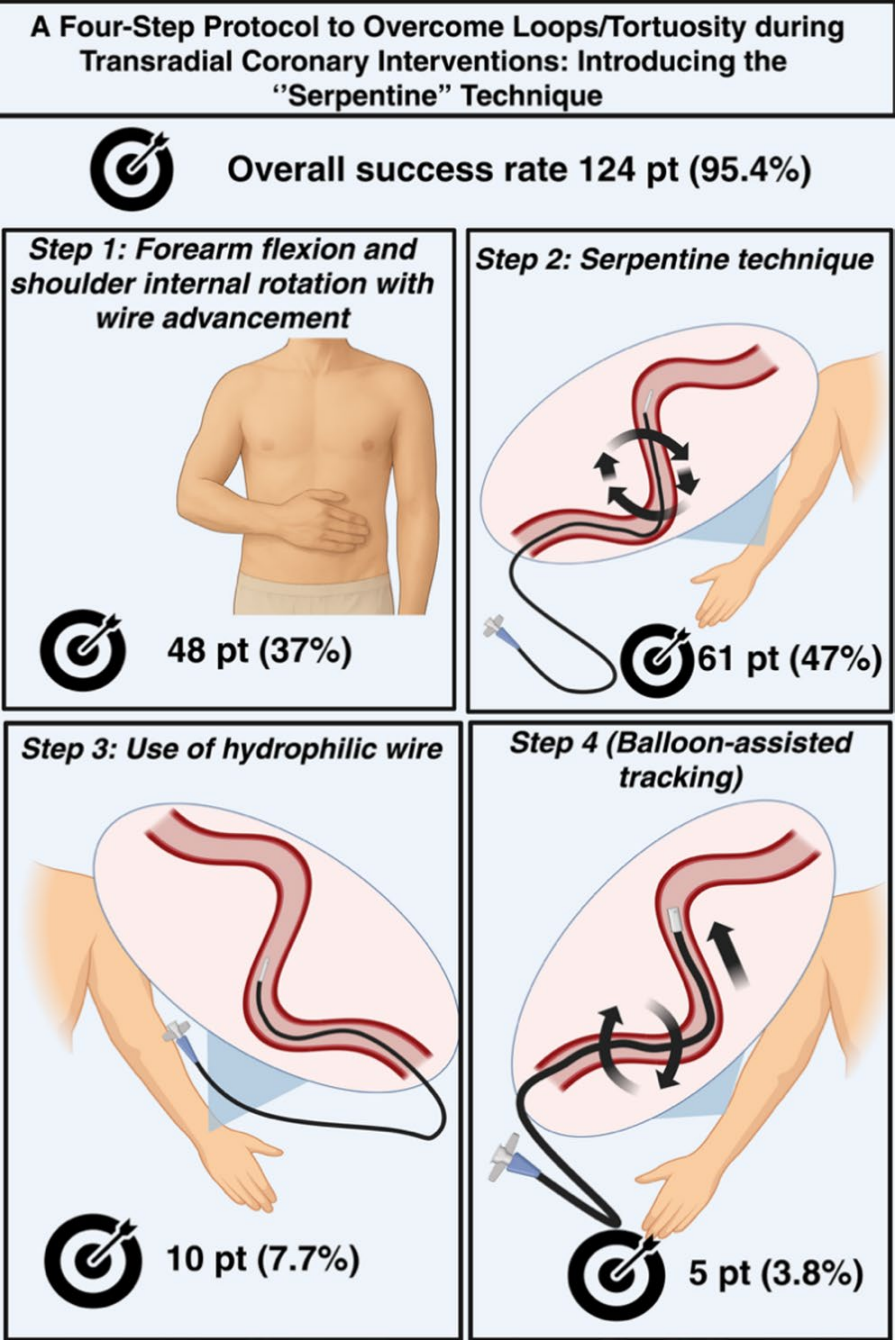

**Supplementary Information:**

The online version contains supplementary material available at 10.1007/s12928-026-01251-9.

## Introduction

In 1989, Lucien Campeau [1] first described transradial access (TRA) for coronary angiography, and in 1993, Ferdinand Kiemeneij [2] introduced the TRA approach for coronary intervention. The use of TRA has steadily increased, while reliance on traditional transfemoral access (TFA) has declined. Current guidelines for revascularization in chronic and acute coronary syndromes (ACS) recommend TRA as the default approach [3, 4], primarily due to its lower complication rates. Two large randomized controlled trials, RIVAL [5] and MATRIX [6], compared TRA with TFA in terms of ischemic outcomes, bleeding, and vascular complications. Both trials demonstrated that TRA is associated with fewer ischemic events [6] and a significant reduction in major bleeding and vascular complications, such as pseudoaneurysms, compared with TFA [5, 6]. Additional benefits include greater cost-effectiveness [7], shorter hospital stays, and earlier ambulation [8, 9]. A large meta-analysis of randomized controlled trials confirmed that the superiority of TRA extends across the full spectrum of coronary artery disease (CAD), including both chronic and acute syndromes [10]. Notably, TRA is now widely used even in complex coronary interventions that were previously performed almost exclusively via TFA. The COLOR trial demonstrated that large-bore TRA for complex procedures—such as chronic total occlusions, left-main interventions, severely calcified lesions, and complex bifurcations—achieved comparable procedural success and ischemic outcomes, while significantly reducing vascular and bleeding complications compared with TFA [11].

Despite these advantages, TRA has certain limitations. It is associated with a slightly longer door-to-balloon time compared with TFA, although this is offset by lower complication rates [12]. The crossover rate remains higher with TRA; however, this can be minimized through the use of the left radial artery (RA), high-volume operator experience, and ultrasound guidance [12]. TRA is also linked to a modest increase in radiation exposure, which can be mitigated in high-volume centers [12]. Conversely, it is associated with lower contrast volume and a reduced risk of contrast-induced acute kidney injury [12].

Anatomical variations and arterial tortuosity are major contributors to procedural failure and increased crossover rates, leading to patient discomfort. Such variations occur in approximately 12–23% of patients [13–15]. Common anomalies include high RA bifurcation, RA loops, RA tortuosity, subclavian tortuosity, and persistent left subclavian artery [13–15]. Among these, RA loops are most frequently associated with procedural failure [13–15]. These anomalies also prolong procedural time, increase radiation exposure, and elevate the risk of vascular complications [16].

Various techniques have been proposed to address these challenges, including manual straightening of loops, use of hydrophilic-coated guidewires and catheters, crossover to ulnar access, torque catheter maneuvers, and balloon- or pigtail-assisted tracking [13, 17–22]. However, no standardized approach for managing radial and brachial artery loops has been established.

This study introduces a structured four-step protocol, termed the “Serpentine” technique, designed to overcome RA loops and facilitate timely, uncomplicated, and successful coronary interventions without the need for crossover.

## Methods

### Patient population

From December 2020 to December 2022, we studied 2,389 patients who underwent TRA coronary angiography (96 cases without PCI) and/or percutaneous coronary intervention (PCI) at our center, performed by three experienced operators (defined as > 300 transradial coronary artery interventions per year). Exclusion criteria included cardiogenic shock, STEMI, absence of right radial pulse, anatomical limitations, and prior CABG. One hundred thirty (5.44%) patients with extreme tortuosity or a loop in the upper arm were included in this prospective, non-randomized study.

An upper arm loop was defined as the presence of a full 360° loop, and extreme tortuosity was defined as the presence of angulation of more than 90° along the vessel.

### Study design

Proximal or distal (at the anatomical snuffbox) RA cannulation was performed with the right arm positioned beside the patient’s body while the wrist was hyperextended without ultrasound guidance. After local anesthesia with 1–2 ml xylocaine 2%, the RA was punctured with a 21-gauge needle from lateral to medial with an angle of approximately 35°. Following guidewire introduction, a 5-Fr sheath (KDL Medical) or a 6-Fr sheath (ARROW International) was inserted at the operator’s discretion. All patients routinely received intra-arterial 200 µg nitroglycerin and unfractionated heparin (50 U/kg for diagnostic catheterization, 100 U/kg for coronary angioplasty), along with a 15 mL normal saline bolus. In cases where advancing the diagnostic catheter was difficult, an angiography of the upper extremity arteries was performed to visualize the vessel anatomy. In cases of extreme tortuosity or looping, our proposed four-step protocol was applied to facilitate catheter advancement.

The study has been reviewed and approved by the local Human Research Ethics Committee. All patients signed informed consent regarding the publication of their data.

### Description of the four-step technique

The protocol is designed to optimize TRA in the presence of loops or tortuosity by first utilizing standard coronary angiography equipment and, when required, implementing advanced catheter manipulation techniques in combination with hydrophilic guidewires and dedicated support devices.

Fluoroscopy was used after successful radial artery cannulation with a standard introducer sheath only when resistance was encountered during advancement of the wire or diagnostic catheter; no routine (upfront) angiography was performed. In such cases, the first step was a careful fluoroscopic survey of the upper arm to identify radial loops or significant tortuosity. When a loop was recognized, a predefined, standardized four-step approach was applied sequentially. No time limits were imposed for completing the sequence, and implementation of the protocol was left to the discretion of the experienced operator managing the case.

#### Step 1

**Fig. 1:**
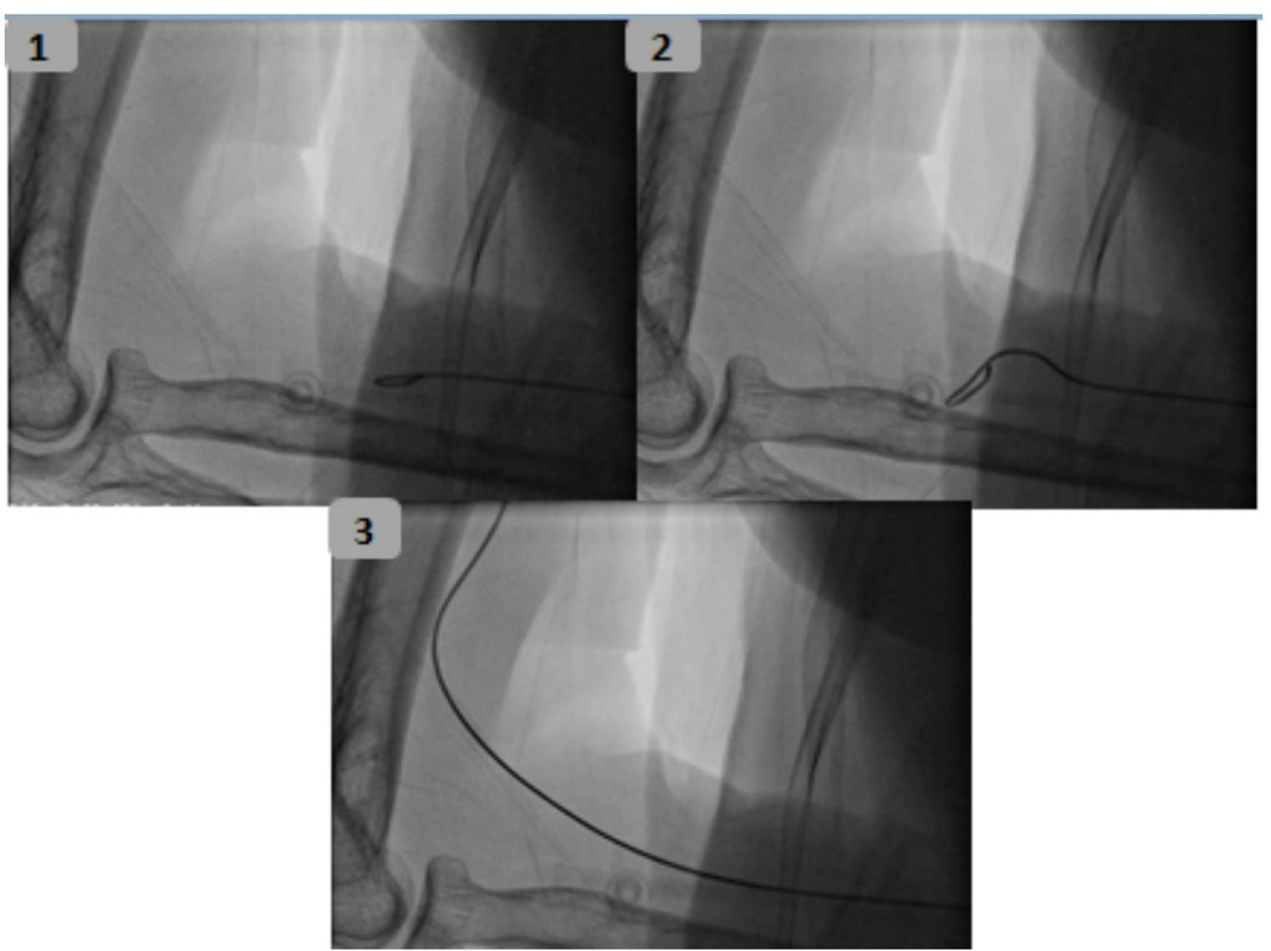
Under fluoroscopic guidance, flex the forearm to 90° and perform concomitant medial rotation of the shoulder while advancing the wire

Under fluoroscopic guidance, flex the forearm to 90° and perform concomitant medial rotation of the shoulder while advancing the wire (Fig. 1). If unsuccessful, or if the tortuosity is located proximal to the cubital fossa, proceed to Step 2.

#### Step 2 (Serpentine Technique)

**Fig. 2:**
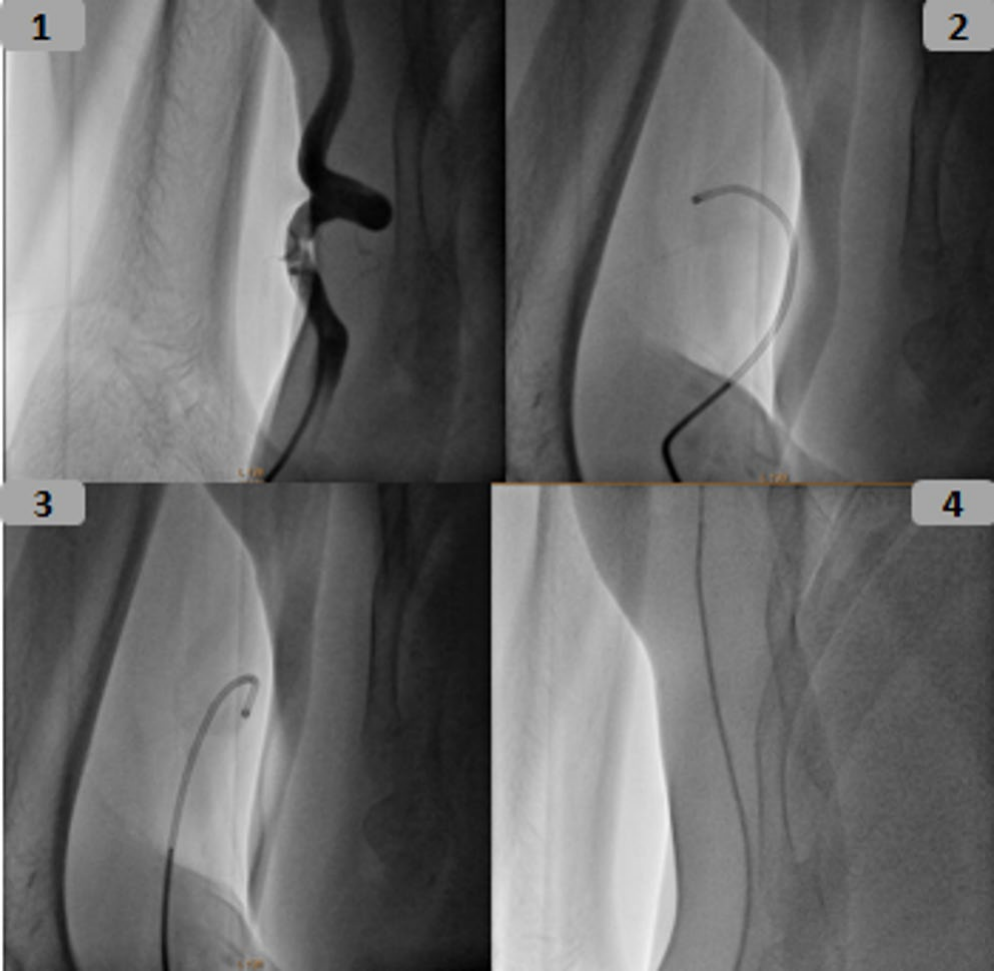
The ‘’Serpentine’’ Technique. Advance the 5-Fr Tiger II diagnostic catheter (TERUMO) using gentle alternating rotational movements to the right and left, while simultaneously retracting the guidewire by 2–3 cm

Advance the 5-Fr Tiger II diagnostic catheter (TERUMO) using gentle alternating rotational movements to the right and left, while simultaneously retracting the guidewire by 2–3 cm (Fig. 2). The key principle of this maneuver is to avoid forceful advancement; instead, the catheter should be manipulated to untwist the loop, like untying a knot. The decision to use the Tiger II universal diagnostic catheter is based on its lower rates of vasospasm, which can help with the successful catheter advancement through loops and tortuosities. However, we cannot safely assume that the curvature of the Tiger II catheter is superior to that of standard Judkins catheters, nor that different curvatures could influence the rate of successful catheter advancement. Yet, the use of a universal catheter reduces the need to exchange catheters, which is particularly important in cases of loops and tortuosities. If this technique is unsuccessful, proceed to Step 3.

#### Step 3

**Fig. 3:**
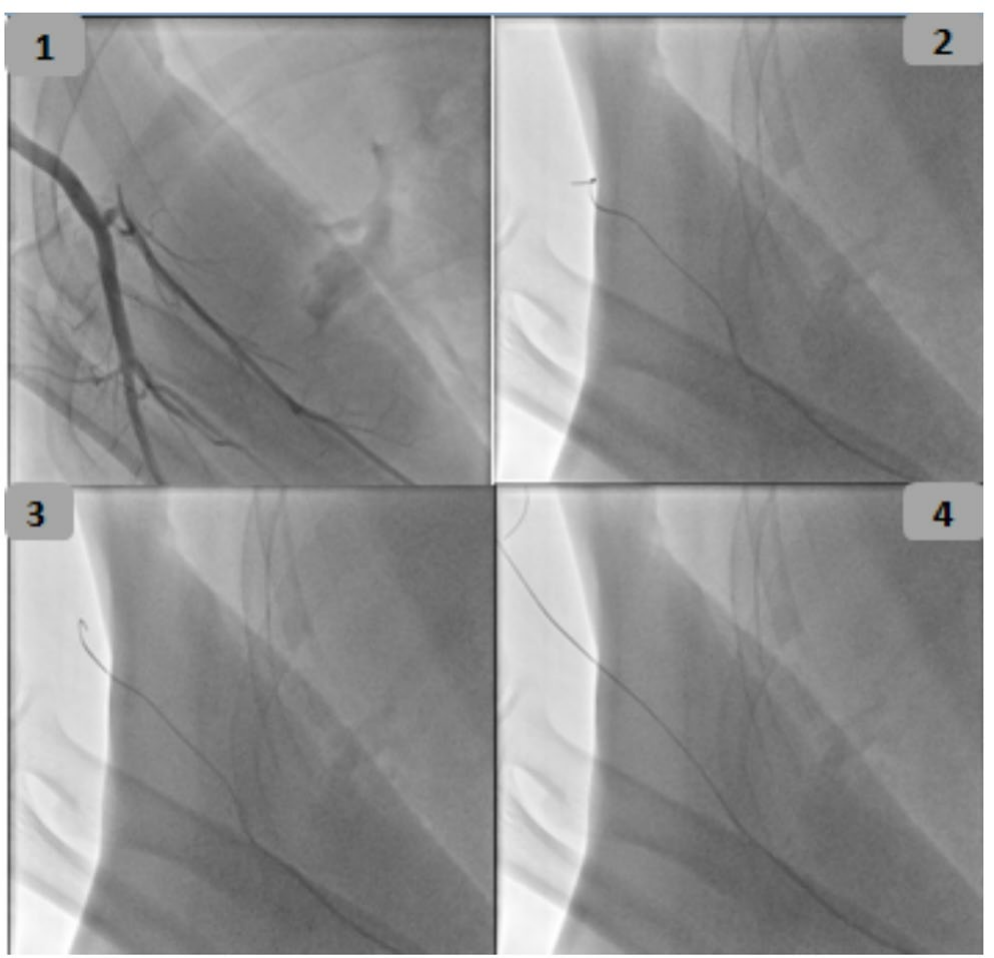
Hydrophilic guidewire. Withdraw the diagnostic catheter approximately 2 cm proximally to the point of resistance and replace the standard wire with a hydrophilic nitinol guidewire (0.035-inch; Cordis Aquatrack)

Withdraw the diagnostic catheter approximately 2 cm proximally to the point of resistance and replace the standard wire with a hydrophilic nitinol guidewire (0.035-inch; Cordis Aquatrack) (Fig. 3). If this step is not successful, proceed to Step 4.

#### Step 4

**Fig. 4:**
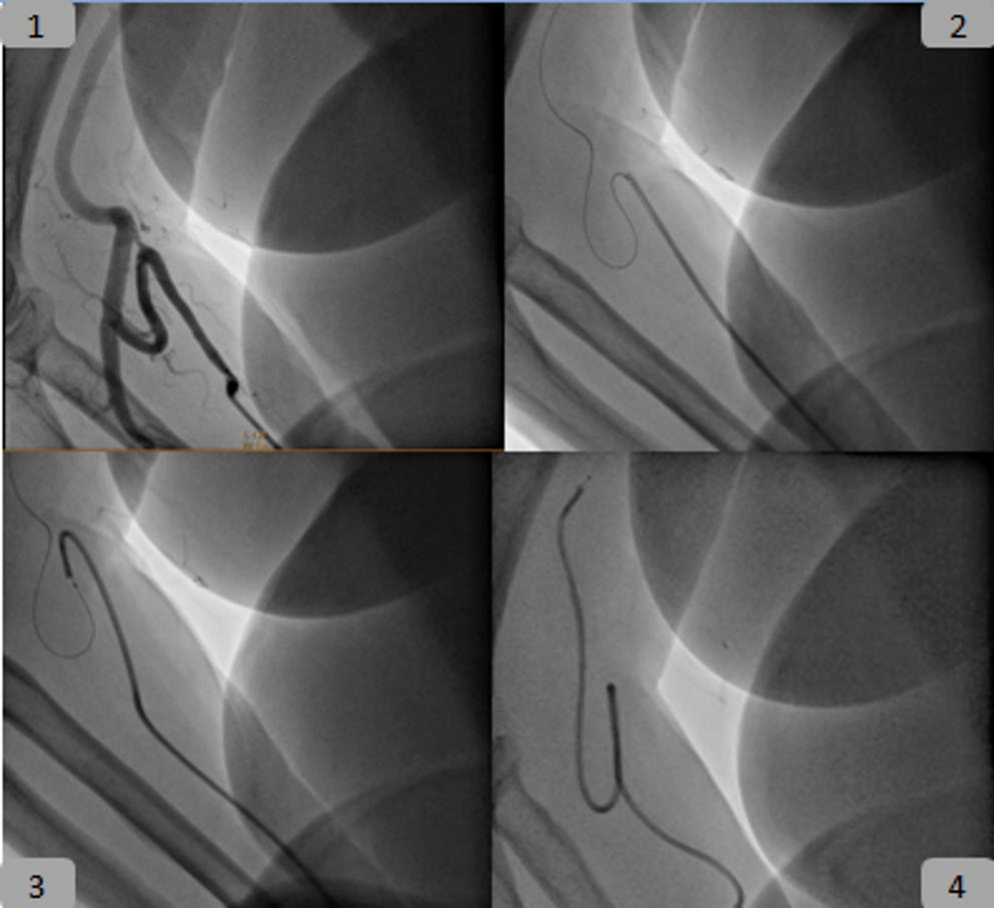
Balloon-Assisted Tracking (BAT) technique. A PTCA balloon is partially advanced beyond the distal tip of the diagnostic catheter, inflated at low pressure (3–6 atm), and the entire assembly is gently advanced over a 0.014-inch soft-tipped PTCA guidewire across the resistant arterial segment in an atraumatic manner

Perform the Balloon-Assisted Tracking (BAT) technique [19]. A PTCA balloon is partially advanced beyond the distal tip of the diagnostic catheter, inflated at low pressure (3–6 atm), and the entire assembly is gently advanced over a 0.014-inch soft-tipped PTCA guidewire across the resistant arterial segment in an atraumatic manner (Fig. 4).

After completion of the coronary angiography, every patient underwent follow-up angiography in order to investigate the vessel anatomy and possible vascular complications (artery dissection, spasm, extravagation etc.).

### Study endpoints

The purpose of our study was to assess the incidence of upper arm tortuosities or loops in patients who underwent coronary intervention at our center and to present a structured four-step protocol, including a novel approach termed the “Serpentine” technique.

The study endpoints included [1] success rate at each step defined as resolution of loop with concomitant safe catheter advancement [2] total procedural success rate defined as completion of the coronary angiography or PCI without need for access-site crossover; [3] necessity for access site crossover; [4] total procedure time- total time duration from upper arm angiography to the successful advancement of the diagnostic catheter to the Valsalva sinuses with the usage of the proposed protocol; [5] total procedural fluoroscopy time during the patient’ stay in the hemodynamic laboratory; [6] access site complications including hematoma, spasm, dissection, bleeding, compartment syndrome, no palpable pulse after, paresis during patient hospitalization.

### Statistical analysis

For the statistical analysis, all continuous variables were reported as the mean and standard deviation (SD) if their distribution was normal; otherwise, the median and interquartile range (IQR) were used. Categorical variables were reported as counts (percentages). Comparisons between groups for categorical variables were performed using Fisher’s exact test when expected counts were less than 5, the chi-square test when expected counts were more than 5, and the Kruskal-Wallis rank sum test for continuous variables.

To identify potential clinical predictors for the successful advancement of the catheter using either ‘Step 1’ or ‘Steps 1 and 2’ of the technique, as well as the total time required for all steps of the technique, we performed logistic regression modelling and linear modelling, respectively. In these sub-analyses, the clinical predictors tested included gender, age, height, weight, diabetes, hypertension, dyslipidemia, smoking, chronic kidney disease, and peripheral arterial disease.

A p-value below 0.05 was regarded as statistically significant. All statistical analyses were conducted using R and R-Studio Version 4.3 (2024).

## Results

### Patients’ baseline characteristics

Patients’ baseline characteristics are shown in Table 1.

**Table 1:** Baseline patient characteristics

	***n*** ** (%)**
Frequency of upper arm loop/ extreme tortuosity	
Total patients	**2389**
Upper arm loop/ extreme tortuosity	**130 (5.44)**
Patient characteristics	
Gender	
Male	**75 (58)**
Female	**55 (42)**
Dyslipidemia	**99 (76)**
Hypertension	**96 (74)**
Diabetes Mellitus	**36 (28)**
Active smoking	**28 (22)**
Prior MI	**22 (17)**
Known stable CAD	**20 (15)**
Heart failure	**17 (13)**
Cerebrovascular Accident	**10 (7.7)**
Chronic Kidney Disease	**9 (6.9)**
Peripheral Arterial Disease	**4 (3.1)**
Dialysis	**1 (0.9)**
Prior PCI	**25 (19)**
Prior right radial artery access	**27 (21)**
Coronary angiography indication	
Unstable angina	
NSTEMI	**45 (35)**
Valvulopathy	**19 (15)**
Stable CAD	**27 (21)**
Heart failure	**15 (12)**
Unknown	**1 (0.9)**

RA= radial artery, loop = a full 360o tortuosity, extreme tortuosity = the presence of angulation > 90o along the vessel, MI= myocardial infarction, CAD= cardiovascular disease, PCI= percutaneous coronary intervention, NSTEMI = non-ST-segment elevation MI- Number of patients (%)

A total of 2,389 patients who underwent coronary angiography at our center were included in our prospective, non-randomized study from December 2020 to December 2022. In 130 patients (5.44%), an extreme tortuosity or loop was found (118 cases in the forearm and 12 cases proximal to the cubital fossa), with a mean age of 74 years, a mean body weight of 77 kg, and a mean height of 166 cm. 75 patients (58%) were men. Also, 28 patients (22%) were active smokers, and 42 (32%) had a previous history of CAD with conservative treatment or PCI. Among well-established comorbidities, the most common was dyslipidemia (76%), followed by hypertension (74%), diabetes mellitus (28%), heart failure (13%), stroke (7.7%), and finally chronic kidney disease (7.8%). 68 (58%) patients presented with unstable angina or NSTEMI, while 19 (15%) patients underwent coronary angiography due to valvulopathy, and 15 (12%) patients due to first diagnosed heart failure. A total of 19 patients (15%) underwent PCI.

### Procedural characteristics

Table 2 presents the procedural characteristics of our 4-step protocol, as well as the procedural complications. With the use of our protocol, successful TRA was achieved in 124 patients (95.4%). Specifically, successful TRA was achieved in 62 (47.7%) patients via right proximal RA and in 61 (46.9%) patients via distal RA, with crossover to an alternative access site required in only 6 cases (4.6%) (2 cases via femoral artery and 4 cases via left RA). The overall rate of successful completion of coronary angiography using our method was 98% (124 cases via right RA and 4 cases via left RA). Notably, in 109 patients (84%), loops or tortuosity were resolved within the first two steps of the protocol.

**Table 2:** Procedural results and complications

Procedural results	
Success Rate of each step	
Step 1	48 (37%)
Step 2	61 (47%)
Step 3	10 (7.7%)
Step 4	5 (3.8%)
Total Procedural Success Rate	124 (95.4%)
Procedural Failure Rate	6 (4.6%)
Total Steps Duration (sec)	115 ± 143 / 60 (30, 120)
Success on step 1	44 ± 23 / 38 (24, 60)
Success on step 2	113 ± 108 / 73 (40, 153)
*With 1 try in step 2*	44 (81.5%)
*With 2 tries in step 2*	9 (17%)
*With 3 tries in step 2*	1 (1.9%)
Success on step 3	257 ± 83 / 255 (200, 318)
Success on step 4	627 ± 160 / 651 (463, 769)
*Fluoroscopy time (sec) without PCI*	226 (131, 404)
Step 1	155 (105, 318)
Step 2	225 (152, 398)
Step 3	323 (255, 855)
Step 4	607 (310, 1017)
Contrast volume (ml)	66 (48, 85)
Step 1	66 (47, 88)
Step 2	62 (49, 79)
Step 3	81 (48, 96)
Step 4	78 (73, 79)
DAP	30,812 (20,948, 44,792)
Step 1	33,083 (21,965, 43,859)
Step 2	29,307 (20,349, 39,611)
Step 3	38,298 (21,549, 58,044)
Step 4	41,592 (33,580, 70,735)
Air Kerma	323 (250, 489)
Step 1	327 (252, 488)
Step 2	314 (246, 439)
Step 3	403 (213, 552)
Step 4	445 (350, 745)
Need for PCI	19 (15%)

Procedural complications	
Pain (> 5)	35 (27%)
Hematoma	5 (3.8%)
Spasm	3 (2.3%)
Dissection	3 (2.3%)
Bleeding	2 (1.5%)
Compartment syndrome	0 (0%)
No palpable pulse before discharge	0 (0%)
Paresis	0 (0%)

Procedural success rate= completion of the coronary angiography without need for access site crossover; Procedural failure rate= need for another access site; Total step duration= total time from the RA angiography to the successful advancement of the diagnostic catheter to the Valsava sinuses with the usage of the proposed protocol; Fluoroscopy time= total procedural fluoroscopy time during the patient’ stay in the hemodynamic laboratory, Pain = > moderate with visual analog pain scale); N(%); mean ± SD; median (IQR)

The median total procedure time was 60 seconds (IQR 30–120), while the mean total procedure time (from upper arm angiography to the successful advancement of the diagnostic catheter to the Valsalva sinuses) was 115 ± 143 seconds with a need for a mean contrast volume of 76.6 ml. Successful passage of the loop at Step 1 or Step 2 was associated with significantly shorter total procedural fluoroscopy times during the patient’ stay in the hemodynamic laboratory [Step 1: 155 s (IQR 105–318); Step 2: 225 s (IQR 152–398)] compared with completion at Step 3 or Step 4 [Step 3: 323 s (IQR 255–855); Step 4: 607 s (IQR 310–1017)] when simple coronary angiography (without PCI) was underwent. On the contrary, successful catheter advancement at Step 1 or Step 2 was not associated with lower radiation exposure, as assessed by DAP and Air Kerma.

### Procedural complications

In terms of complications, no patients developed compartment syndrome or paresis. Bleeding was evident in 2 cases (1.5%), and all cases were BARC Type 1 bleedings. Also, 5 patients (3.8%) developed a hematoma (2 cases were presented with type Ia hematoma, 1 case with type Ib hematoma, 1 case with type II hematoma, 1 case with type III hematoma according to the modified EASY classification); though we did not document during which step of the protocol each hematoma occurred. Pain (defined as at least moderate with a visual analog pain scale) at the access site was evident in 35 cases (27%). In all patients, the RA pulse was palpable after the coronary angiography.

### Predictors of procedural success and total procedure time

To assess for factors that influence the success of our protocol, and more specifically during step 1 or step 2, along with the total procedure time, we conducted a univariate analysis to assess the predictive effect of clinical factors. No significant associations were identified between the evaluated clinical variables and procedural outcomes. Supplementary Table 1 summarizes the results of the univariate analysis assessing predictors of procedural success at Step 1 and after Steps 1 + 2, as well as total procedure time, according to various clinical characteristics.

## Discussion

In previous studies, various techniques have been proposed to address the anatomical variations of the RA and facilitate the process of coronary angiography, including manual straightening of loops [18], the BAT technique [20–22], the use of a hydrophilic coated guidewire, the ‘’Knuckle’’ technique, and the usage of a microcatheter [17–22]. In this study, we present a reproducible, user-friendly protocol for interventional cardiologists that incorporates commonly used equipment and strategies along with a novel maneuver—the “Serpentine’’ Technique—to manage upper arm loops and severe tortuosity. Using this protocol, the success rate of TRA without crossover was 95.4%, and the overall rate of successful coronary angiography completion was 98%. While the “Serpentine” technique appears similar to other torque-assisted techniques and to microcatheter- or hydrophilic wire-dependent techniques, the novelty of the “Serpentine” technique lies to the fact that it does not require exchange of catheters and wire, since the initial universal Tiger II diagnostic catheter is advanced through the guidewire; thus, it leads to significant reduction in the total procedural time in most of the cases, given that almost 84% of the loops are tackled with Step 1 and 2 and that subsequently we can proceed with catheterization of both left and right coronary arteries with no additional need of catheter exchange.

In patients with ACS, TRA—strongly recommended by current guidelines—requires substantial operator and institutional experience to optimize outcomes. Moreover, TRA for neuroangiographic procedures is increasing in popularity, and its effectiveness and safety have been proven in several studies [23, 24]. High-volume operators and centers are associated with significantly shorter fluoroscopy time, lower contrast volume, and reduced PCI failure rates. Intrinsic characteristics of the RA, including its small caliber and the higher prevalence of tortuosity, loops, and vasospasm, remain the primary causes of TRA failure [14–22]. In our study, the incidence of upper arm loops and tortuosity was 5.44%, which aligns with findings from other published series [14–17, 25–28] that showed an estimated incidence in the general population ranging from 3.3 to 7.1%.

Especially, regarding upper arm tortuosities or loops, there is no widely accepted technique to overcome these anatomical variations, which are the main cause of TRA failure, with a crossover rate ranging from 14.2 to 39% [14, 15]. Notably, in a series of 650 Egyptian patients, the presence of an upper arm loop was associated with an 87.5% procedural failure rate. In contrast, upper arm tortuosity was linked to a 28.5% failure rate [15]. The methods used to address this significant problem include manual straightening of the loop [18], the use of a hydrophilic wire [13], and the BAT technique [20–22]. The use of such techniques, especially the BAT technique, increases the procedure success rates from 69.5% to 98.3% [20–22]. Another series reported a 100% success rate with the use of the BAT technique [20]. However, these techniques involve exchanging equipment and using additional wires and balloons. The main advantage of our structured 4-step protocol is that it proposes two simple maneuvers (step 1 with forearm flexion and step 2 with the “Serpentine” technique), which do not require any exchange of wires and catheters, and are associated with an approximately 84% success rate.

Another crucial feature that needs to be addressed is that our technique is beneficial for reducing total procedure time and fluoroscopy time. Notably, the use of the BAT technique is associated with a significant increase in the total procedure time. The series of cases by Felekos et al. reported a total of 131.2 ± 41.3 s added procedural time due to the use of the BAT technique [20]. In our study, the total added time due to BAT was even longer [median 324 s (IQR 293, 359)]. Additionally, the need for wire exchange and the use of a hydrophilic wire (step 3) is associated with a median added time of 82 s (IQR 30, 115). The major strength of our 4-step protocol is its median procedural time of 60 s (IQR 30, 120), with approximately 84% of cases manageable in just two simple and fast steps. Additionally, the use of our 4-step protocol is associated with a significant reduction in fluoroscopy time, even though there is no significant difference in DAP and Air Kerma.

Finally, the use of our 4-step protocol was safe, as no major vascular or access-site complications were observed. Specifically, the proposed “Serpentine” technique, combined with the use of a hydrophilic wire and BAT, did not result in perforation or compartment syndrome. In our study, only a few minor procedural complications were reported. These findings align with other series, which show that techniques for managing loops and tortuosities are generally safe [13, 17–22].

The proposed four-step protocol, incorporating the “Serpentine” technique, is straightforward to learn and enables near-complete procedural success, even in cases with severe upper arm loops and tortuosity, with a crossover rate of only 4.6%. Furthermore, this approach significantly reduces total procedure time and fluoroscopy exposure without increasing procedural complications—an advantage of particular importance in primary PCI for ACS patients and other complex coronary interventions.

## Future directions

Future directions may include the integration of advanced imaging technologies or the development of novel catheter designs to further facilitate navigation through upper arm loops. Additionally, creating specialized guidewires or sheaths tailored for loop management could help overcome these procedural challenges.

## Conclusion

According to findings of this study, the four-step strategy provides a structured approach for managing upper arm loops, incorporating the “Serpentine” technique to overcome severe tortuosity. This method enhances procedural success, shortens overall procedure and fluoroscopy times, and minimizes the risk of complications during RA catheterization.

## Limitations

This study has some limitations. It was performed at a single center by three highly experienced operators from the same department, which may limit broader generalizability. We did not systematically capture the total number of forearm or upper-arm angiographies performed specifically in response to resistance during wire or catheter advancement, and operator-level outcomes were not available to explore potential operator-related differences in success, radiation exposure, or complications. Ultrasound guidance was not used. Radial artery patency was assessed only during hospitalization, so late radial artery occlusion could not be evaluated. In addition, we did not record the time from skin puncture to successful catheter advancement, an important measure of procedural efficiency. Finally, because of routine documentation constraints, we were unable to perform a detailed analysis of how procedural and anatomical factors (e.g., access site, loop/tortuosity location, or angulation severity) might influence early-step success or overall procedure time. While the proposed protocol yielded encouraging results, further validation—ideally in prospective, multicenter comparative studies—would help define its clinical utility.

## Impact on clinical practice

Anatomical arterial variations, and especially upper arm loops/tortuosity, form a common clinical problem during coronary interventions via TRA. As mentioned above, various techniques have been proposed to address these challenges, but unfortunately, no standardized approach for managing them has been established. Our study introduces a structured four-step protocol designed to address upper arm artery loops, thereby improving procedural efficiency, safety, time duration, and patient outcomes.

## Supplementary Information

Below is the link to the electronic supplementary material.

Supplementary Material 1

Supplementary Material 2

Supplementary Material 3

Supplementary Material 4

Supplementary Material 5

## Abbreviations

RA: Radial Artery
TRA: Transradial Access
TFA: Transfemoral Access
CABG: Coronary Artery Bypass Grafting
NSTEMI: Non-ST-Elevation Myocardial Syndrome
STEMI: ST-Elevation Myocardial Syndrome
ACS: Acute Coronary Syndromes
CAD: Coronary Artery Disease
PCI: Percutaneous Coronary Intervention
BAT: Balloon-Assisted Tracking Technique
