# Supplementary material for: A four-step protocol to overcome loops/tortuosity during transradial coronary interventions: introducing the ‘’Serpentine’’ technique
Source: Cardiovasc Interv Ther. 2026 Mar 21;41(3):586–95. doi: 10.1007/s12928-026-01251-9 (PMC13279476; doi:10.1007/s12928-026-01251-9)
Supplement: Supplementary file 1 — Supplementary Material 1 [file 12928_2026_1251_MOESM1_ESM.docx]

# Supplementary data

**A Four-Step Protocol to Overcome Loops/Tortuosity during Transradial Coronary Interventions: Introducing the ‘’Serpentine’’ Technique**

# Running title: The ‘’Serpentine’’ Protocol and Technique

**Authors**

Grigorios Tsigkas^1^, MD, PhD; Nikoleta Kalovrenti^1^, MD, PhD(c); Spyridon Graidis^1^, MD, MSc; Michail Papafaklis^1^, MD, PhD; Dimitrios Chlorogiannis^1^, MD; Athanasios Moulias^1^, MD, PhD; Georgios Vasilagkos^1^, MD, PhD(c); Eleni-Evangelia Koufou^1^, MD, PhD; Panagiota Spyropoulou^1^, MD; Nikolaos Vythoulkas^1^ MD; Nikolaos Kartas^1^ MD; Periklis Davlouros^1^, MD, PhD

**Affiliations**

1. Cardiology Department, University Hospital of Patras, Patras, Greece

**Address for correspondence**

Associate Professor Grigorios Tsigkas, MD, PhD

Address: Rion, 26504, Patras, Greece

Tel: +302613603836

| Supplementary Table 1. Univariate analysis for prediction of procedural success and total procedure time | | | |
| --- | --- | --- | --- |
| *Characteristic* | *Success in Step 1*  *p-value* | *Success in Step 1+2*  *p-value* | *Total procedure time*  *p-value* |
| Female | 0.91 | 0.956 | 0.931 |
| Age | 0.33 | 0.915 | 0.408 |
| Height | 0.124 | 0.507 | 0.860 |
| Weight | 0.163 | 0.394 | 0.755 |
| PAD | 0.62 | 0.63 | 0.711 |
| DM | 0.906 | 0.145 | 0.59 |
| Hypertension | 0.55 | 0.783 | 0.536 |
| Dyslipidemia | 0.508 | 0.997 | 0.737 |
| Smoking | 0.555 | 0.157 | 0.907 |
| CKD | 0.629 | 0.999 | 0.369 |
| Dialysis | 1 | 1 | 0.247 |
| Prior R radial | 0.664 | 0.832 | 0.687 |
| Aspirin | 0.804 | 0.349 | 0.124 |
| P2Y12i | 0.731 | 0.315 | 0.798 |
| Anticoagulants | 0.687 | 0.339 | 0.881 |
| ACEi/ARBs | 0.952 | 0.725 | 0.464 |
| B-blockers | 0.731 | 0.123 | 0.287 |
| CCBs | 0.206 | 0.769 | 0.227 |
| Nitrates | 0.478 | 0.949 | 0.158 |
| Diuretics | 0.656 | 0.497 | 0.136 |
| Statins | 0.192 | 0.612 | 0.427 |

Supplementary table 1. Univariate analysis for prediction of procedural success and total procedure time. Assessment of predictors for procedural success at Step 1 and after Steps 1 + 2, and for predictors of total procedure time, according to various clinical characteristics.

## Moving images

**Moving image 1**. 90° flexion of the forearm with concomitant medial rotation of the shoulder while advancing the wire, under fluoroscopic guidance.

**Moving image 2. Serpentine Technique**: Advancement of the 5-Fr Tiger II diagnostic catheter (TERUMO) using gentle alternating rotational movements to the right and left, while simultaneously retracting the guidewire by 2–3 cm and avoiding forceful advancement; instead, the catheter should be manipulated to untwist the loop, like untying a knot.

**Moving image 3**. Withdrawal of the diagnostic catheter approximately 2 cm proximally to the point of resistance and replacement of the standard wire with a hydrophilic nitinol guidewire (0.035-inch; Cordis Aquatrack).

**Moving image 4. Balloon-Assisted Tracking (BAT) technique**. Partial advancement of a PTCA balloon beyond the distal tip of the diagnostic catheter, inflated at low pressure (3–6 atm), and gentle advancement of the entire over a 0.014-inch soft-tipped PTCA guidewire across the resistant arterial segment in an atraumatic manner.
